# Changes in Physical Fitness among Elementary and Middle School Students in Korea before and after COVID-19

**DOI:** 10.3390/ijerph191811712

**Published:** 2022-09-16

**Authors:** Eui-Jae Lee, Dong-il Seo, Seung-Man Lee, Jong-Hyuck Kim

**Affiliations:** 1 Department of Physical Education, Graduate School of Education, Sogang University, Seoul 04107, Korea; 2 Department of Health and Exercise Science, Dongguk University, Gyeongju-si 38066, Korea; 3 Department of Physical Education, College of Education, Korea University, Seoul 02841, Korea; 4 Department of Medical Beauty Care, Jungwon University, Goesan-gun 28024, Korea

**Keywords:** COVID-19, elementary school students, middle school students, physical activity, physical activity promotion system

## Abstract

The present study aimed to analyze changes in health-related physical fitness among Korean elementary and middle school students before (2019) and after (2021) the COVID-19 pandemic. Data collection was completed by requesting the physical activity promotion system (PAPS) data from elementary and middle school students. This information is obtained annually by the Goyang Office of Education in Gyeonggi-do, Korea. The collected data were measured in 2019 and 2021. Data were collected from 17,000 children in the fifth and sixth grades of elementary school and about 24,000 boys and girls in the first, second, and third grades of middle school. Chi-square analyses were used to examine data from each school’s health-related physical fitness examinations. Our results indicated that physical fitness levels were significantly lower in 2021 than in 2019 across the following six areas: cardiorespiratory endurance, power, muscular strength, flexibility, obesity, and overall health-related physical fitness (*p* < 0.05). In addition, the ratio of students with excellent physical fitness (PAPS Grades 1 and 2) significantly decreased from 2019 to 2021, while the ratio of students with poor physical fitness (PAPS Grades 3, 4, and 5) increased (*p* < 0.05). In addition, there were some differences according to grade and gender. Discussions regarding the impact of decreases in physical activity on physical fitness, interpretations of physical fitness in the context of a pandemic, and practical measures that can be implemented to improve health and fitness among children and adolescents in such situations remain essential.

## 1. Introduction

The COVID-19 pandemic altered many aspects of daily life. In particular, decreases in physical activity during the pandemic exerted a significant impact on respiratory, cardiovascular, and musculoskeletal health [1]. While such changes critically impacted adults, they equally affected elementary and middle school students. Notably, face-to-face physical education classes were discontinued in favor of online classes to ensure adherence to social distancing measures and prevent the spread of COVID-19 [2,3].

Analyzing 29 studies related to various physical activities during the COVID-19 pandemic, Polero et al. [4] reported a lack of evidence upon which to base guidelines for exercise or adapt physical education within the context of COVID-19. The American College of Sports Medicine recommends at least 30 min of vigorous exercise at least five days a week [5]. This has been practically impossible during the COVID-19 pandemic, given the restrictions and prohibitions on outdoor activities in 2020 and 2021. Various studies conducted in Korea, Spain, and the United States have reported that both adults and adolescents have faced difficulty engaging in outdoor physical activities during the pandemic, and overall levels of physical activity have decreased given the difficulty of engaging in physical activity at home [4,6,7].

Physical activity during adolescence plays a key role in shaping a healthy lifestyle, thus providing important contributions to current and future mental and physical health [8,9,10]. Despite the importance of physical education in promoting health maintenance during adolescence, interruptions in physical education have been difficult to prevent during COVID-19. Research has demonstrated that students’ physical fitness levels decreased during the pandemic [11,12,13,14]. Specifically, a study on physical fitness changes in the midst of the COVID-19 situation was conducted on students in Japan, China, and Austria. Most students reported that their physical fitness levels were decreasing [15,16,17,18]. Moreover, various studies have examined educational, in addition to physical, aspects that have changed because of the COVID-19 situation. Kamila and Mróz [18] reported that Polish university students continued to feel anxiety and uncertainty and had difficulty learning at home through remote classes and organizing what they learned, as assessed by taking tests. Asanov et al. [19] surveyed 1500 high school students in Ecuador concerning how much they participated in distance learning during the COVID-19 pandemic. More than 74% of the students participated in distance learning, but students who faced social isolation experienced increased depression. Sait and Guveli [20] found that families receiving free meals, single-parent families, and homes with parents who had limited education in the UK spent much less time studying at home. Further, they reported that students should have time to study through offline and online remote classes. As such, many studies have examined how to operate students’ remote education in the context of COVID-19, in addition to assessing the severity of the current situation and how to return to more traditional forms of education after the COVID-19 situation is over [21,22,23].

Based on previous studies, various educational and psychological problems have been found, in addition to the loss of physical activity among elementary and middle school students in the COVID-19 situation. Studies [4,12] have shown that physical fitness levels have decreased, but how physical fitness levels have specifically changed has not been examined. Nor have there been any large-scale studies on elementary and middle school students. Considering this gap in the existing research, this study attempted to analyze changes in health-related physical fitness before and after the COVID-19 pandemic for elementary and middle school students in Korea using data obtained through the physical fitness promotion system (PAPS). Since 2010, all public and private schools in Korea have participated in the PAPS, which aims to evaluate physical fitness and provide exercise-related guidance to elementary, middle, and high school students based on assessments of physical factors such as power, flexibility, cardiorespiratory endurance, muscle endurance, and body composition [24,25]. The impact of PAPS has been assessed by various studies. For instance, Song et al. [25] examined the effect of providing students with various exercise programs on obesity and physical fitness. Lee et al. [24] reported improvements in physical fitness at a one-year follow-up assessment when high school students were provided morning exercise programs.

The present study aimed to investigate changes in health-related physical fitness levels among elementary and middle school students following the emergence of COVID-19 (from 2019 to 2021).

## 2. Materials and Methods

### 2.1. Participants

This study utilized 2019 and 2021 data obtained from elementary (5th and 6th grades; aged 12–13 years) and middle school students (1st, 2nd, and 3rd grades of middle school; aged 14–16 years) enrolled in Goyang, Gyeonggi-do, Korea.

Elementary and middle schools in Korea are required to participate in the PAPS every year and report their results to the Korea Office of Education. These data are used to investigate students’ health and physical conditions. In this study, data were collected by requesting the yearly PAPS data from the person in charge of the Office of Education. The collected data were from 80 elementary and 38 middle schools. About 17,000 data sets were obtained from the 5th- and 6th-grade students in 2019 and 2021, and about 24,000 data sets were obtained from students in their 1st, 2nd, and 3rd years of middle school. The students who participated in the PAPS in 2019 and 2021 were selected as study participants because complete data did not exist for 2020. Because of the severity of COVID-19, not every school measured their students’ fitness with the PAPS. It is important to note that the data collected by the Korea Office of Education does not include personal information from the students, nor is the school’s name displayed.

We obtained the relevant data after receiving approval from the Gyeonggi-do Office of Education. This study was performed in accordance with the guidelines of the Declaration of Helsinki and was approved by the Gyeonggi Provincial Office of Education (2022-02). Since personal information such as name, telephone number, social security number, and address was not collected, ethical approval was not required. The number of study participants for each assessment in 2019 and 2021 are shown in Table 1.

### 2.2. Assessment Variables

Five physical fitness variables were measured: cardiorespiratory endurance, power, muscular strength, flexibility, and obesity. Cardiorespiratory endurance was assessed via a shuttle run test. This test measures the number of times students make a round trip (15 m for elementary and 20 m for middle school) before the timed music runs out. Power was assessed via a standing long jump test. This test measures the distance jumped (in cm) from a standing position, without a starting run. Muscular strength was assessed based on the results of a grip strength test. Grip force is measured in kilograms for both the right and left hand using a grip machine. Level of flexibility was assessed using a sit-and-reach test. The curve was measured in cm by bending the upper body based on the baseline, which left a forward curve measured in a seated state [15]. Obesity status was determined based on body mass index, which was calculated using each participant’s height and weight [16]. Students’ physical fitness levels were measured based on their overall health and physical fitness scores by adding scores in five areas. The rating criteria for each physical fitness factor were different for the young men and women in all grades, as shown in Table 2.

Within each area of assessment, fitness levels were classified from Grade 1 (best) to 5 (worst). Body composition was divided into five categories: Grade 1 (normal), Grade 2 (underweight), Grade 3 (overweight), Grade 4 (mild obesity), and Grade 5 (severe obesity). An overall health-related physical fitness score was determined for each student based on the scores for each of the five assessments.

### 2.3. Data Processing

Data were analyzed using SPSS 18.0 (IBM Corp., Armonk, NY, USA). First, frequency analysis was conducted to examine differences according to gender, grade, and year. After classifying participants according to gender, grade, and year, chi-square analysis was performed to examine changes in cardiorespiratory endurance, power, muscular strength, flexibility, obesity, and overall health-related physical fitness from 2019 to 2021, thus representing the changes since the emergence of COVID-19. The level of statistical significance was set at 0.05.

## 3. Results

### 3.1. Cardiorespiratory Endurance

In Table 3, chi-square analysis revealed a significant difference in cardiorespiratory endurance scores between 2019 and 2021. Among elementary school students (in fifth and sixth grades) of both genders, we observed a significant reduction in the proportion of Grades 1 and 2 and a significant increase in the proportion of Grades 4 and 5. In addition, the proportion of Grades 1, 2, and 3 combined decreased among middle school students, while the proportion of Grades 3, 4, and 5 increased. In particular, rates of excellent endurance were lower among middle school boys compared with elementary school boys (middle school: first 9.1%, second 15.8%, third 19.9%) and among middle school girls compared with elementary school girls (middle school: first 14.2%, second 15.8%, third 12.3%).

### 3.2. Power

In Table 4, chi-square analysis also revealed significant differences in power before and after COVID-19 among fifth-grade girls and sixth-grade boys. In addition, significant differences in power were observed among both boys and girls at all middle school levels when comparing data between 2019 and 2021. In particular, compared with boys, fewer girls achieved grades of 1 and 2 in 2021 than in 2019. In the case of female students in their first year of middle school, such scores decreased by 11.3%. In the second grade, they decreased by 8.3%, and in the third grade, they decreased by 8.6%. Meanwhile, the frequency of Grades 3, 4, and 5 among girls significantly increased from 2019 to 2021 compared with boys.

### 3.3. Muscular Strength

In Table 5, chi-square analysis revealed significant differences in muscular strength between 2019 and 2021. Differences were observed between boys and girls in all grades except the last year of elementary and the first year of middle school. However, the frequencies of Grades 1 and 2 did not decrease by more than 10% for cardiorespiratory endurance or power, and there were no significant differences according to grade between 2019 and 2021.

### 3.4. Flexibility

In Table 6, chi-square analysis revealed significant differences in flexibility grades between 2019 and 2021. No significant differences were observed among fifth-grade girls, second-year middle school girls, or boys and girls in the third year of middle school. In addition, the χ^2^ value was the highest for male students in the sixth grade, at 76.239; however, this difference was not significant when compared with those for other physical factors.

### 3.5. Obesity Status

In Table 7, chi-square analysis revealed significant differences in obesity grades between 2019 and 2021, except among girls in the third year of middle school. In particular, obesity frequency increased among elementary and middle school students due to a decrease in the frequency of normal-weight/thin grades and an increase in the ratio of overweight, mild obesity, and severe obesity grades.

### 3.6. Overall Health-Related Physical Fitness Scores

In Table 8, among both elementary and middle school students, significant differences in the overall health-related physical fitness scores were observed for both boys and girls between 2019 and 2021. Our analysis indicated that the frequency of Grades 1 and 2 decreased, while the frequency of Grades 3 and 4 increased. In particular, there was a greater difference among middle school than elementary school students. The x2 values for boys and girls in the second year of middle school and for boys in the third year were 344.394, 226.981, and 251.844, respectively, indicating large differences in overall health and fitness scores between 2019 and 2021.

## 4. Discussion

The present study aimed to analyze changes in health-related physical fitness among elementary and middle school students in Korea before (2019) and after (2021) the COVID-19 pandemic. Our results indicated that physical fitness levels were lower among both elementary and middle school students after the COVID-19 pandemic.

### 4.1. Interpretation of Findings

Looking at the results for the changes in cardiorespiratory endurance, both elementary and middle school students saw a decrease in Grades 1 and 2 and an increase in Grades 3, 4, and 5. In particular, in the case of male students in the third year of middle school, the change in physical fitness was the largest. The first grade was 41.4% in 2019, but it decreased by about 20% to 21.5% in 2021. In addition, in the case of female students, the ratio of the first grade decreased the most, from 36.2% to 20.4%. Due to COVID-19, both elementary and middle school students were unable to go to school and learned through online education. Consequently, external activities were cut off and physical activity opportunities were reduced, as the results of previous studies [14,26] showed. After the WHO’s pandemic declaration, the scope of life for students was very narrow. In particular, the amount of physical activity students participated in decreased when school was restricted. The decrease in students’ cardiorespiratory endurance can be attributed to this decrease in activity.

Next, looking at the results for the change in power, elementary school students did not show as much difference as they did for other physical fitness factors, but power decreased for both men and women in all three grades of middle school. On the other hand, it increased for both genders in the third, fourth, and fifth grades of elementary school. In particular, compared with males, females in their first year of middle school had a higher rate of decrease. In a study by Pinho et al. [27], male students’ power rapidly decreased due to COVID-19. Moreover, the power of elementary and middle school students in Korea continuously decreased compared with 2019. These results can also be interpreted as a decrease in students’ physical activity due to the pandemic situation. Since power is a combination of innate motor sense and planned training, planned physical activities are necessary to improve it. In particular, it is worth noting that female students’ power decreased when regular physical activities were not provided in physical education classes at school. Because male students are relatively interested in sports events and have a strong desire to move, their access to and participation in various physical activities is higher than that of female students [24]. The results derived from this study confirmed that the physical activity program provided by the school was essential to maintain and improve the balanced power among female students.

Third, compared with other physical factors, there was no significant change in muscular strength for either elementary or middle school students. The chi-square values for 2019 and 2021 were not significant, nor did they show a sharp decrease in muscular strength. A study by Sagarra-Romero and Viñas-Barros [28] found that physical activity decreased rapidly for the elderly during the COVID-19 pandemic, and the decrease in muscular strength was significant. In addition, in a study by Young et al. [29], similar results to ours were found, in that a decrease in physical activity among the elderly and other adults did not lead to significant changes in muscular strength. Muscular strength is developed, in part, through basic movements and exercises that can be done at home during the course of our daily lives. Therefore, outside physical activity changes may not have much effect. Online learning due to the pandemic restricted physical education classes. However, Korean physical education teachers have made great efforts to increase students’ physical activity in online-based classes. In particular, the expansion of home training centered on muscular strength and muscle endurance has become popular nationwide. These findings on muscular strength indicate maintained muscular strength, of course. Alternatively, they demonstrate the persistence of its power, and they may be interpreted to show how home training during online class periods as a result of the pandemic did not reduce students’ muscular strength, as derived in this study.

Fourth, overall changes in flexibility decreased in Grades 1 and 2 in 2021. Especially noticeable were the changes seen in both male and female students in the final year of elementary school and the first year in middle school. A previous study by Song et al. [25] reported significant improvement in flexibility (pre = 2.64 ± 3.49, post = 5.98 ± 2.78) through a 16-week health exercise program geared toward adolescents. Lee et al. [24] also reported improved flexibility after a one-year physical activity program. Bartolo et al.’s [30] study reported that trunk-specific rehabilitation treatment for 4 weeks improves the lateral trunk flexion of Parkinson’s patients, showing that there was no decrease in flexibility due to reduced physical activity. Flexibility can be improved through static and dynamic stretching. Health management is an important content element in the Korean physical education curriculum. In particular, stretching before and after class plays a major role in improving students’ flexibility. This stretching was inevitably limited in the pandemic situation. The decrease in flexibility after the pandemic can be interpreted as a reflection of these limitations. For adolescents whose physical activity has been reduced due to COVID-19, specific measures are needed to improve youth flexibility by providing them with a suitable exercise program [14,27].

Finally, the results for changes in obesity were very different from those for other physical factors. In particular, obesity increased mainly among male students in the fifth and sixth years of elementary school and the first, second, and third years of middle school. This is consistent with the results of previous studies showing that obesity naturally increased due to decreased physical activity [31,32]. In addition, Lange et al. [33] investigated the obesity levels of adolescents in the U.S. during the COVID-19 situation, and the number of obese adolescents increased during the COVID-19 period. As discussed above, there were changes in cardiorespiratory endurance, power, muscular strength, and flexibility before and after the pandemic declaration, but the decreased obesity rate was more dramatic than any other observed change. In general, in addition to the basal metabolic rate, people use energy when participating in physical activity. If the nutrients they consume are constant and physical activity decreases, the obesity rate increases. These results highlight the changes that much of our society have experienced because of the physical restrictions imposed by government-controlled “social distancing” aimed at preventing the spread of an infectious disease, as was the case in the unprecedented special situation of COVID-19. Schools, places of education where many people come in contact daily, were not untouched by the pandemic situation. In particular, physical education, which is centered on physical activity, was inevitably hit hard. In the end, it can be interpreted that students’ decreased physical activity naturally resulted in an increase in obesity.

Several studies have highlighted the need to address decreased physical activity since the outbreak of COVID-19 in 2020, especially among children, as this decrease has persisted following the pandemic situation [33]. In particular, researchers have stressed the importance of high-intensity, rather than general low-intensity, physical activity [34,35].

### 4.2. Practical Implications of the Study

Recently reclassified as endemic, COVID-19 has become a part of daily life. As such, there is a great need for discussion regarding the types of physical education offered to elementary and middle school students in this context. Normal school operations were significantly inhibited by COVID-19, and teachers experienced numerous difficulties due to a lack of familiarity with implementing online physical education classes [3,34]. In particular, given limitations in terms of space and the availability of sports equipment, online physical education classes consisted mainly of training exercises that could be performed at home [36]. Several reports have suggested that online physical education classes have been relatively ineffective for children [37,38,39]. Although face-to-face physical education classes have mostly resumed, physical education teachers must play a key role in promoting and advancing basic physical education classes at the elementary and middle school levels. Kim et al. [38] reported several technical difficulties related to the implementation of online classes, citing that physical education during the COVID-19 pandemic was most effective when offered face-to-face rather than online. As such, the role of physical education teachers is considered important in an endemic situation [38,39,40].

In addition to ensuring regular implementation of physical education classes, expanding opportunities to participate in various sports activities beyond those associated with regular classes may help to improve physical fitness levels among elementary and middle school students. School sports clubs are common in Korea [41,42,43], allowing students to select a preferred activity in which to engage during lunch and before/after school. However, participation in such clubs was largely restricted during the COVID-19 pandemic [44], with even the Olympic Games being postponed by a year. Nevertheless, some physical education teachers in Korea attempted to operate school sports club events online [44,45]. Although participation in ball sports such as soccer and basketball was not possible, these teachers have been able to provide limited physical activity despite social distancing measures. While most Korean schools are now able to conduct classes as usual, additional efforts are required to ensure that sports activities and competitions are reintroduced along with physical education classes.

One major limitation of the current study is that it remains difficult to generalize our findings, given that the results were derived from assessments conducted among elementary and middle school students in Korea. That is, changes in physical activity levels may have differed across countries due to differences in the effects of COVID-19 and the different preventive measures utilized. Therefore, further studies are required to examine how the COVID-19 pandemic has impacted health and fitness levels in each country. Another limitation is that participation in other activities could not be controlled. Moreover, this study used chi-square and frequency analysis for the sake of reliability. Further well-designed statistical methods, such as linear regression analysis, are necessary to provide an even more detailed analytical explanation of these and similar data so that a broader understanding of adolescent physical fitness can be achieved.

## 5. Conclusions

Our results demonstrated that physical fitness levels decreased among both elementary and middle school students in Korea from 2019 to 2021. Further research is required to identify the most effective strategies for improving physical fitness among these students, given that COVID-19 has become endemic in Korea.

## Figures and Tables

**Table 1:** Number of study participants between 2019 and 2021.

Grade	Gender		Cardiorespiratory Endurance	Power	Muscular Strength	Flexibility	Obesity	Overall Health-Related Physical Fitness Score
Elementary school (5th grade)	Boys	2019	4429	4429	4429	4766	4321	4433
		2021	4716	4754	4770	4429	4338	4774
	Girls	2019	4248	4250	4252	4542	4146	4257
		2021	4499	4532	4543	4250	4147	4547
Elementary school (6th grade)	Boys	2019	4589	4569	4591	4632	4510	4601
		2021	4578	4627	4635	4592	4470	4643
	Girls	2019	4493	4386	4396	4288	4335	4417
		2021	4251	4289	4292	4408	4148	4299
Middle school (1st grade)	Boys	2019	4264	4200	4255	4769	4267	4277
		2021	4670	4740	4721	4269	4737	4776
	Girls	2019	4202	4190	4182	4621	4199	4208
		2021	4486	4589	4581	4201	4599	4634
Middle school (2nd grade)	Boys	2019	4201	4181	4198	4951	4165	4202
		2021	4958	4942	4823	4200	4783	5066
	Girls	2019	4085	4069	4086	4755	4056	4092
		2021	4759	4743	4631	4083	4587	4781
Middle school (3rd grade)	Boys	2019	4671	4669	4637	4509	4671	4685
		2021	4526	4508	4491	4669	4519	4452
	Girls	2019	4514	4512	4481	4432	4509	4523
		2021	4447	4427	4404	4509	4442	4452

**Table 2:** Measurement standard table.

**Grade**	**Gender**	**Shuttle Run Test (Repetitions)** **Elementary School 15 m Middle School 20 m**					**Standing Long Jump (cm)**				
		**Grade 5**	**Grade 4**	**Grade 3**	**Grade 2**	**Grade 1**	**Grade 5**	**Grade 4**	**Grade 3**	**Grade 2**	**Grade 1**
Elementary school (5th grade)	Boys	28 under	29–49	50–72	73–99	100 up	111 under	111.1–141	141.1–159	159.1–180	180.1 up
	Girls	22 under	23–44	45–62	63–84	85 up	100 under	100.1–123	123.1–139	139.1–170	170.1 up
Elementary school (6th grade)	Boys	31 under	32–53	54–77	78–103	104 up	122 under	122.1–148	148.1–167	167.1–200	200.1 up
	Girls	24 under	25–49	50–68	69–92	93 up	100 under	100.1–127	127.1–144	144.1–175	175.1 up
Middle school (1st grade)	Boys	19 under	20–35	36–49	50–63	64 up	131 under	131.1–159	159.1–177	177.1–211	211.1 up
	Girls	13 under	14–18	19–24	25–34	35 up	100 under	100.1–127	127.1–144	144.1–175	175.1 up
Middle school (2nd grade)	Boys	21 under	22–37	38–51	52–65	66 up	136 under	136.1–169	169.1–187	187.1–218	218.1 up
	Girls	14 under	15–20	21–28	29–39	40 up	100 under	100.1–127	127.1–145	145.1–183	183.1 up
Middle school (3rd grade)	Boys	23 under	24–39	40–53	54–67	68 up	145 under	145.1–180	180.1–201	201.1–238	238.1 up
	Girls	15 under	16–22	23–32	33–44	45 up	100 under	100.1–127	127.1–145	145.1–183	183.1 up
		**Hand Grip Strength (kg)**					**Sit and Reach (cm)**				
		**Grade 5**	**Grade 4**	**Grade 3**	**Grade 2**	**Grade 1**	**Grade 5**	**Grade 4**	**Grade 3**	**Grade 2**	**Grade 1**
Elementary school (5th grade)	Boys	12.4 under	12.5–16.9	17.0–22.9	23.0–30.9	31.0 up	−4.1 under	−4.0–0.9	1.0–4.9	5.0–7.9	8.0 up
	Girls	11.9 under	12.0–15.4	15.5–18.9	19.0–28.9	29.0 up	0 under	0.1–4.9	5.0–6.9	7.0–9.9	10.0 up
Elementary school (6th grade)	Boys	14.9 under	15.0–18.9	19.0–26.4	26.5–34.9	35.0 up	−4.1 under	−4.0–0.9	1.0–4.9	5.0–7.9	8.0 up
	Girls	13.9 under	14.0–18.9	19.0–21.9	22.0–32.9	33.0 up	1.9 under	2.0–4.9	5.0–9.9	10.0–13.9	14.0 up
Middle school (1st grade)	Boys	16.4 under	16.5–22.4	22.5–29.9	30.0–41.9	42.0 up	−4.1 under	−4.0–1.9	2.0–5.9	6.0–9.9	10.0 up
	Girls	13.9 under	14.0–18.9	19.0–22.9	23.0–35.9	36.0 up	1.9 under	2.0–7.9	8.0–10.9	11.0–14.9	15.0 up
Middle school (2nd grade)	Boys	21.9 under	22.0–28.4	28.5–36.9	37.0–44.4	44.5 up	−4.1 under	−4.0–1.9	2.0–6.9	7.0–9.9	10.0 up
	Girls	13.9 under	14.0–19.4	19.5–25.4	25.5–35.9	36.0 up	1.9 under	2.0–7.9	8.0–10.9	11.0–14.9	15.0 up
Middle school (3rd grade)	Boys	24.9 under	25.0–32.9	33.0–40.4	40.5–48.4	48.5 up	−3.1 under	−3.0–2.5	2.6–6.9	7.0–9.9	10.0 up
	Girls	15.9 under	16.0–19.4	19.5–27.4	27.5–35.9	36.0 up	1.9 under	2.0–7.9	8.0–10.9	11.0–15.9	16.0 up
		**Body Mass Index (kg/m^2^)**					**Health-Related Physical Fitness (Total Score)**				
		**Grade 1** **(Thin)**	**Grade 2** **(Normal)**	**Grade 3** **(Overweight)**	**Grade 4** **(Mild obesity)**	**Grade 5** **(Severe obesity)**	**Grade 5**	**Grade 4**	**Grade 3**	**Grade 2**	**Grade 1**
Elementary school (5th grade)	Boys	14.3 under	14.4–20.9	21.0–23.3	23.4–33.3	33.4 up	19 under	20–39	40–59	60–79	80 up
	Girls	14.6 under	14.7–21.7	21.8–24.4	24.5–34.5	34.6 up					
Elementary school (6th grade)	Boys	14.9 under	15.0–21.7	21.8–24.0	24.1–34.0	34.1 up					
	Girls	15.0 under	15.1–22.3	22.4–25.1	25.2–35.2	35.3 up					
Middle school (1st grade)	Boys	15.4 under	15.5–22.6	22.7–24.7	24.8–34.7	34.8 up					
	Girls	15.5 under	15.6–22.9	23.0–25.7	25.8–35.8	35.9 up					
Middle school (2nd grade)	Boys	16.1 under	16.2–23.1	23.2–25.6	25.7–35.6	35.7 up					
	Girls	15.9 under	16.0–23.3	23.4–26.2	26.3–36.3	36.4 up					
Middle school (3rd grade)	Boys	16.7 under	16.8–23.7	23.8–25.9	26.0–35.9	36.0 up					
	Girls	16.5 under	16.6–23.7	23.8–26.6	26.7–36.7	36.8 up					

**Table 3:** Comparison of cardiorespiratory endurance in 2019 and 2021.

Grade	Gender			Grade 1	Grade 2	Grade 3	Grade 4	Grade 5	Total	χ^2^/*p*
Elementary school (5th grade)	Boys	2019	Frequency	1190	1108	1413	555	163	4429	χ^2^ = 355.740 df = 4 *p* < 0.001
			Percentage	26.9%	25.0%	31.9%	12.5%	3.7%	100.0%	
		2021	Frequency	775	980	1519	961	480	4715	
			Percentage	16.4%	20.8%	32.2%	20.4%	10.2%	100.0%	
	Girls	2019	Frequency	1026	1217	1374	555	76	4248	χ^2^ = 302.505 df = 4 *p* < 0.001
			Percentage	24.2%	28.6%	32.3%	13.1%	1.8%	100.0%	
		2021	Frequency	703	1046	1467	1047	236	4499	
			Percentage	15.6%	23.2%	32.6%	23.3%	5.2%	100.0%	
Elementary school (6th grade)	Boys	2019	Frequency	1209	1214	1355	642	169	4589	χ^2^ = 319.111 df = 4 *p* < 0.001
			Percentage	26.3%	26.5%	29.5%	14.0%	3.7%	100.0%	
		2021	Frequency	814	952	1380	950	482	4578	
			Percentage	17.8%	20.8%	30.1%	20.8%	10.5%	100.0%	
	Girls	2019	Frequency	877	1210	1503	693	120	4403	χ^2^ = 254.372 df = 4 *p* < 0.001
			Percentage	19.9%	27.5%	34.1%	15.7%	2.7%	100.0%	
		2021	Frequency	559	956	1368	1093	275	4251	
			Percentage	13.1%	22.5%	32.2%	25.7%	6.5%	100.0%	
Middle school (1st grade)	Boys	2019	Frequency	1081	799	1013	1086	285	4264	χ^2^ = 294.060 df = 4 *p* < 0.001
			Percentage	25.4%	18.7%	23.8%	25.5%	6.7%	100.0%	
		2021	Frequency	759	672	995	1575	669	4670	
			Percentage	16.3%	14.4%	21.3%	33.7%	14.3%	100.0%	
	Girls	2019	Frequency	1805	1097	794	346	160	4202	χ^2^ = 268.535 df = 4 *p* < 0.001
			Percentage	43.0%	26.1%	18.9%	8.2%	3.8%	100.0%	
		2021	Frequency	1309	1126	1062	626	363	4486	
			Percentage	29.2%	25.1%	23.7%	14.0%	8.1%	100.0%	
Middle school (2nd grade)	Boys	2019	Frequency	1481	734	937	726	323	4201	χ^2^ = 428.352 df = 4 *p* < 0.001
			Percentage	35.3%	17.5%	22.3%	17.3%	7.7%	100.0%	
		2021	Frequency	969	664	1270	1380	675	4958	
			Percentage	19.5%	13.4%	25.6%	27.8%	13.6%	100.0%	
	Girls	2019	Frequency	1478	925	947	480	255	4085	χ^2^ = 380.416 df = 4 *p* < 0.001
			Percentage	36.2%	22.6%	23.2%	11.8%	6.2%	100.0%	
		2021	Frequency	969	941	1387	948	514	4759	
			Percentage	20.4%	19.8%	29.1%	19.9%	10.8%	100.0%	
Middle school (3rd grade)	Boys	2019	Frequency	1936	883	904	628	320	4671	χ^2^ = 527.925 df = 4 *p* < 0.001
			Percentage	41.4%	18.9%	19.4%	13.4%	6.9%	100.0%	
		2021	Frequency	974	751	1248	1036	517	4526	
			Percentage	21.5%	16.6%	27.6%	22.9%	11.4%	100.0%	
	Girls	2019	Frequency	1193	1133	1205	662	321	4514	χ^2^ = 375.202 df = 4 *p* < 0.001
			Percentage	26.4%	25.1%	26.7%	14.7%	7.1%	100.0%	
		2021	Frequency	628	876	1403	866	674	4447	
			Percentage	14.1%	19.7%	31.5%	19.5%	15.2%	100.0%	

**Table 4:** Comparison of power in 2019 and 2021.

Grade	Gender			Grade 1	Grade 2	Grade 3	Grade 4	Grade 5	Total	χ^2^/*p*
Elementary school (5th grade)	Boys	2019	Frequency	462	1212	1347	1262	146	4429	χ^2^ = 22.939 df = 4 *p* = 0.011
			Percentage	10.4%	27.4%	30.4%	28.5%	3.3%	100.0%	
		2021	Frequency	487	1239	1459	1318	251	4754	
			Percentage	10.2%	26.1%	30.7%	27.7%	5.3%	100.0%	
	Girls	2019	Frequency	293	1494	1243	1071	149	4250	χ^2^ = 13.0905 df = 4 *p* < 0.001
			Percentage	6.9%	35.2%	29.2%	25.2%	3.5%	100.0%	
		2021	Frequency	330	1637	1286	1062	217	4532	
			Percentage	7.3%	36.1%	28.4%	23.4%	4.8%	100.0%	
Elementary school (6th grade)	Boys	2019	Frequency	403	1537	1406	1044	179	4569	χ^2^ = 29.143 df = 4 *p* < 0.001
			Percentage	8.8%	33.6%	30.8%	22.8%	3.9%	100.0%	
		2021	Frequency	385	1452	1416	1085	289	4627	
			Percentage	8.3%	31.4%	30.6%	23.4%	6.2%	100.0%	
	Girls	2019	Frequency	506	1498	1313	932	137	4386	χ^2^ = 8.633 df = 4 *p* = 0.071
			Percentage	11.5%	34.2%	29.9%	21.2%	3.1%	100.0%	
		2021	Frequency	435	1476	1302	905	171	4289	
			Percentage	10.1%	34.4%	30.4%	21.1%	4.0%	100.0%	
Middle school (1st grade)	Boys	2019	Frequency	583	1550	1182	832	53	4200	χ^2^ = 292.876 df = 4 *p* < 0.001
			Percentage	13.9%	36.9%	28.1%	19.8%	1.3%	100.0%	
		2021	Frequency	401	1518	1256	1219	346	4740	
			Percentage	8.5%	32.0%	26.5%	25.7%	7.3%	100.0%	
	Girls	2019	Frequency	861	1748	882	586	113	4190	χ^2^ = 314.634 df = 4 *p* < 0.001
			Percentage	20.5%	41.7%	21.1%	14.0%	2.7%	100.0%	
		2021	Frequency	421	1785	1164	1042	177	4589	
			Percentage	9.2%	38.9%	25.4%	22.7%	3.9%	100.0%	
Middle school (2nd grade)	Boys	2019	Frequency	760	1562	956	781	122	4181	χ^2^ = 72.385 df = 4 *p* < 0.001
			Percentage	18.2%	37.4%	22.9%	18.7%	2.9%	100.0%	
		2021	Frequency	740	1687	1122	1124	269	4942	
			Percentage	15.0%	34.1%	22.7%	22.7%	5.4%	100.0%	
	Girls	2019	Frequency	700	1592	880	708	189	4069	χ^2^ = 176.032 df = 4 *p* < 0.001
			Percentage	17.2%	39.1%	21.6%	17.4%	4.6%	100.0%	
		2021	Frequency	424	1769	1213	1126	211	4743	
			Percentage	8.9%	37.3%	25.6%	23.7%	4.4%	100.0%	
Middle school (3rd grade)	Boys	2019	Frequency	727	1850	1092	851	149	4669	χ^2^ = 252.967 df = 4 *p* < 0.001
			Percentage	15.6%	39.6%	23.4%	18.2%	3.2%	100.0%	
		2021	Frequency	351	1487	1243	1205	222	4508	
			Percentage	7.8%	33.0%	27.6%	26.7%	4.9%	100.0%	
	Girls	2019	Frequency	770	1749	934	879	180	4512	χ^2^ = 159.032 df = 4 *p* < 0.001
			Percentage	17.1%	38.8%	20.7%	19.5%	4.0%	100.0%	
		2021	Frequency	419	1608	1221	932	247	4427	
			Percentage	9.5%	36.3%	27.6%	21.1%	5.6%	100.0%	

**Table 5:** Comparison of muscular strength in 2019 and 2021.

Grade	Gender			Grade 1	Grade 2	Grade 3	Grade 4	Grade 5	Total	χ^2^/*p*
Elementary school (5th grade)	Boys	2019	Frequency	400	687	1996	1160	186	4429	χ^2^ = 20.223 df = 4 *p* = 0.011
			Percentage	9.0%	15.5%	45.1%	26.2%	4.2%	100.0%	
		2021	Frequency	393	843	2181	1110	243	4770	
			Percentage	8.2%	17.7%	45.7%	23.3%	5.1%	100.0%	
	Girls	2019	Frequency	360	1198	1416	1026	252	4252	χ^2^ = 59.370 df = 4 *p* < 0.001
			Percentage	8.5%	28.2%	33.3%	24.1%	5.9%	100.0%	
		2021	Frequency	360	1558	1535	848	242	4543	
			Percentage	7.9%	34.3%	33.8%	18.7%	5.3%	100.0%	
Elementary school (6th grade)	Boys	2019	Frequency	550	842	2125	837	237	4591	χ^2^ = 30.885 df = 4 *p* < 0.001
			Percentage	12.0%	18.3%	46.3%	18.2%	5.2%	100.0%	
		2021	Frequency	427	994	2159	791	264	4635	
			Percentage	9.2%	21.4%	46.6%	17.1%	5.7%	100.0%	
	Girls	2019	Frequency	444	1278	1252	1203	219	4396	χ^2^ = 65.703 df = 4 *p* = 0.071
			Percentage	10.1%	29.1%	28.5%	27.4%	5.0%	100.0%	
		2021	Frequency	255	1470	1204	1162	201	4292	
			Percentage	5.9%	34.2%	28.1%	27.1%	4.7%	100.0%	
Middle school (1st grade)	Boys	2019	Frequency	540	1289	1495	777	154	4255	χ^2^ = 30.581 df = 4 *p* < 0.001
			Percentage	12.7%	30.3%	35.1%	18.3%	3.6%	100.0%	
		2021	Frequency	761	1440	1611	725	184	4721	
			Percentage	16.1%	30.5%	34.1%	15.4%	3.9%	100.0%	
	Girls	2019	Frequency	529	1718	1272	583	80	4182	χ^2^ = 8.638 df = 4 *p* = 0.071
			Percentage	12.6%	41.1%	30.4%	13.9%	1.9%	100.0%	
		2021	Frequency	507	1902	1395	662	115	4581	
			Percentage	11.1%	41.5%	30.5%	14.5%	2.5%	100.0%	
Middle school (2nd grade)	Boys	2019	Frequency	844	875	1430	791	258	4198	χ^2^ = 24.552 df = 4 *p* < 0.001
			Percentage	20.1%	20.8%	34.1%	18.8%	6.1%	100.0%	
		2021	Frequency	888	978	1875	802	280	4823	
			Percentage	18.4%	20.3%	38.9%	16.6%	5.8%	100.0%	
	Girls	2019	Frequency	687	1288	1582	481	48	4086	χ^2^ = 46.668 df = 4 *p* < 0.001
			Percentage	16.8%	31.5%	38.7%	11.8%	1.2%	100.0%	
		2021	Frequency	755	1407	1733	582	154	4631	
			Percentage	16.3%	30.4%	37.4%	12.6%	3.3%	100.0%	
Middle school (3rd grade)	Boys	2019	Frequency	1006	1048	1429	950	204	4637	χ^2^ = 21.510 df = 4 *p* < 0.001
			Percentage	21.7%	22.6%	30.8%	20.5%	4.4%	100.0%	
		2021	Frequency	812	1084	1447	916	232	4491	
			Percentage	18.1%	24.1%	32.2%	20.4%	5.2%	100.0%	
	Girls	2019	Frequency	730	1143	2178	338	92	4481	χ^2^ = 87.872 df = 4 *p* < 0.001
			Percentage	16.3%	25.5%	48.6%	7.5%	2.1%	100.0%	
		2021	Frequency	522	1067	2171	453	191	4404	
			Percentage	11.9%	24.2%	49.3%	10.3%	4.3%	100.0%	

**Table 6:** Comparison of flexibility in 2019 and 2021.

Grade	Gender			Grade 1	Grade 2	Grade 3	Grade 4	Grade 5	Total	χ^2^/*p*
Elementary school (5th grade)	Boys	2019	Frequency	2463	826	931	325	221	4766	χ^2^ = 32.469 df = 4 *p* = 0.011
			Percentage	51.7%	17.3%	19.5%	6.8%	4.6%	100.0%	
		2021	Frequency	2034	852	934	361	248	4429	
			Percentage	45.9%	19.2%	21.1%	8.2%	5.6%	100.0%	
	Girls	2019	Frequency	3126	589	321	328	178	4542	χ^2^ = 4.584 df = 4 *p* = 0.333
			Percentage	68.8%	13.0%	7.1%	7.2%	3.9%	100.0%	
		2021	Frequency	2870	607	315	308	150	4250	
			Percentage	67.5%	14.3%	7.4%	7.2%	3.5%	100.0%	
Elementary school (6th grade)	Boys	2019	Frequency	2445	738	816	405	228	4632	χ^2^ = 76.239 df = 4 *p* < 0.001
			Percentage	52.8%	15.9%	17.6%	8.7%	4.9%	100.0%	
		2021	Frequency	2017	819	980	470	306	4592	
			Percentage	43.9%	17.8%	21.3%	10.2%	6.7%	100.0%	
	Girls	2019	Frequency	2377	800	703	199	209	4288	χ^2^ = 39.023 df = 4 *p* < 0.001
			Percentage	55.4%	18.7%	16.4%	4.6%	4.9%	100.0%	
		2021	Frequency	2197	828	826	280	277	4408	
			Percentage	49.8%	18.8%	18.7%	6.4%	6.3%	100.0%	
Middle school (1st grade)	Boys	2019	Frequency	2101	917	866	627	258	4769	χ^2^ = 35.113 df = 4 *p* < 0.001
			Percentage	44.1%	19.2%	18.2%	13.1%	5.4%	100.0%	
		2021	Frequency	1649	867	896	561	296	4269	
			Percentage	38.6%	20.3%	21.0%	13.1%	6.9%	100.0%	
	Girls	2019	Frequency	2583	793	483	521	241	4621	χ^2^ = 22.407 df = 4 *p* < 0.001
			Percentage	55.9%	17.2%	10.5%	11.3%	5.2%	100.0%	
		2021	Frequency	2147	769	493	561	231	4201	
			Percentage	51.1%	18.3%	11.7%	13.4%	5.5%	100.0%	
Middle school (2nd grade)	Boys	2019	Frequency	2340	672	993	589	357	4951	χ^2^ = 20.160 df = 4 *p* < 0.001
			Percentage	47.3%	13.6%	20.1%	11.9%	7.2%	100.0%	
		2021	Frequency	1830	562	891	532	385	4200	
			Percentage	43.6%	13.4%	21.2%	12.7%	9.2%	100.0%	
	Girls	2019	Frequency	2731	716	435	563	310	4755	χ^2^ = 6.7895 df = 4 *p* = 0.148
			Percentage	57.4%	15.1%	9.1%	11.8%	6.5%	100.0%	
		2021	Frequency	2264	686	398	478	257	4083	
			Percentage	55.4%	16.8%	9.7%	11.7%	6.3%	100.0%	
Middle school (3rd grade)	Boys	2019	Frequency	2242	525	715	642	385	4509	χ^2^ = 7.042 df = 4 *p* = 0.134
			Percentage	49.7%	11.6%	15.9%	14.2%	8.5%	100.0%	
		2021	Frequency	2205	576	782	717	389	4669	
			Percentage	47.2%	12.3%	16.7%	15.4%	8.3%	100.0%	
	Girls	2019	Frequency	2431	800	389	485	327	4432	χ^2^ = 3.981 df = 4 *p* = 0.409
			Percentage	54.9%	18.1%	8.8%	10.9%	7.4%	100.0%	
		2021	Frequency	2396	834	395	544	340	4509	
			Percentage	53.1%	18.5%	8.8%	12.1%	7.5%	100.0%	

**Table 7:** Comparison of obesity status in 2019 and 2021.

Grade	Gender			Grade 1 (Normal)	Grade 2 (Thin)	Grade 3 (Overweight)	Grade 4 (Mild Obesity)	Grade 5 (Severe Obesity)	Total	χ^2^/*p*
Elementary school (5th grade)	Boys	2019	Frequency	2829	116	823	509	44	4321	χ^2^ = 137.579 df = 4 *p* < 0.001
			Percentage	65.5%	2.7%	19.0%	11.8%	1.0%	100.0%	
		2021	Frequency	2379	92	973	797	97	4338	
			Percentage	54.8%	2.1%	22.4%	18.4%	2.2%	100.0%	
	Girls	2019	Frequency	3061	168	558	347	12	4146	χ^2^ = 93.752 df = 4 *p* < 0.001
			Percentage	73.8%	4.1%	13.5%	8.4%	0.3%	100.0%	
		2021	Frequency	2738	128	695	559	27	4147	
			Percentage	66.0%	3.1%	16.8%	13.5%	0.7%	100.0%	
Elementary school (6th grades)	Boys	2019	Frequency	3000	107	697	613	93	4510	χ^2^ = 105.354 df = 4 *p* < 0.001
			Percentage	66.5%	2.4%	15.5%	13.6%	2.1%	100.0%	
		2021	Frequency	2537	105	836	817	175	4470	
			Percentage	56.8%	2.3%	18.7%	18.3%	3.9%	100.0%	
	Girls	2019	Frequency	3193	133	585	391	33	4335	χ^2^ = 19.801 df = 4 *p* < 0.001
			Percentage	73.7%	3.1%	13.5%	9.0%	0.8%	100.0%	
		2021	Frequency	2927	96	646	442	37	4148	
			Percentage	70.6%	2.3%	15.6%	10.7%	0.9%	100.0%	
Middle school (1st grade)	Boys	2019	Frequency	2996	117	460	595	99	4267	χ^2^ = 177.183 df = 4 *p* < 0.001
			Percentage	70.2%	2.7%	10.8%	13.9%	2.3%	100.0%	
		2021	Frequency	2750	125	612	997	253	4737	
			Percentage	58.1%	2.6%	12.9%	21.0%	5.3%	100.0%	
	Girls	2019	Frequency	3223	120	529	276	51	4199	χ^2^ = 29.283 df = 4 *p* < 0.001
			Percentage	76.8%	2.9%	12.6%	6.6%	1.2%	100.0%	
		2021	Frequency	3319	130	672	397	81	4599	
			Percentage	72.2%	2.8%	14.6%	8.6%	1.8%	100.0%	
Middle school (2nd grade)	Boys	2019	Frequency	2924	105	274	665	197	4165	χ^2^ = 100.200 df = 4 *p* < 0.001
			Percentage	70.2%	2.5%	6.6%	16.0%	4.7%	100.0%	
		2021	Frequency	2889	129	382	1040	343	4783	
			Percentage	60.4%	2.7%	8.0%	21.7%	7.2%	100.0%	
	Girls	2019	Frequency	3106	91	438	359	62	4056	χ^2^ = 23.399 df = 4 *p* < 0.001
			Percentage	76.6%	2.2%	10.8%	8.9%	1.5%	100.0%	
		2021	Frequency	3383	157	497	436	114	4587	
			Percentage	73.8%	3.4%	10.8%	9.5%	2.5%	100.0%	
Middle school (3rd grade)	Boys	2019	Frequency	3339	154	156	760	262	4671	χ^2^ = 84.236 df = 4 *p* < 0.001
			Percentage	71.5%	3.3%	3.3%	16.3%	5.6%	100.0%	
		2021	Frequency	2835	156	192	989	347	4519	
			Percentage	62.7%	3.5%	4.2%	21.9%	7.7%	100.0%	
	Girls	2019	Frequency	3470	145	366	422	106	4509	χ^2^ = 6.013 df = 4 *p* = 0.198
			Percentage	77.0%	3.2%	8.1%	9.4%	2.4%	100.0%	
		2021	Frequency	3395	170	339	408	130	4442	
			Percentage	76.4%	3.8%	7.6%	9.2%	2.9%	100.0%	

**Table 8:** Comparison of overall health-related physical fitness in 2019 and 2021.

Grade	Sex			Grade 1	Grade 2	Grade 3	Grade 4	Grade 5	Total	χ^2^/*p*
Elementary school (5th grade)	Boys	2019	Frequency	207	1468	2165	546	47	4433	χ^2^ = 125.701 df = 4 *p* < 0.001
			Percentage	4.7%	33.1%	48.8%	12.3%	1.1%	100.0%	
		2021	Frequency	156	1261	2349	923	85	4774	
			Percentage	3.3%	26.4%	49.2%	19.3%	1.8%	100.0%	
	Girls	2019	Frequency	203	1775	1947	321	11	4257	χ^2^ = 86.797 df = 4 *p* < 0.001
			Percentage	4.8%	41.7%	45.7%	7.5%	0.3%	100.0%	
		2021	Frequency	155	1620	2202	532	38	4547	
			Percentage	3.4%	35.6%	48.4%	11.7%	0.8%	100.0%	
Elementary school (6th grade)	Boys	2019	Frequency	267	1565	2114	618	37	4601	χ^2^ = 141.978 df = 4 *p* < 0.001
			Percentage	5.8%	34.0%	45.9%	13.4%	0.8%	100.0%	
		2021	Frequency	224	1123	2377	836	83	4643	
			Percentage	4.8%	24.2%	51.2%	18.0%	1.8%	100.0%	
	Girls	2019	Frequency	197	1675	2118	406	21	4417	χ^2^ = 25.069 df = 4 *p* < 0.001
			Percentage	4.5%	37.9%	48.0%	9.2%	0.5%	100.0%	
		2021	Frequency	174	1463	2144	481	37	4299	
			Percentage	4.0%	34.0%	49.9%	11.2%	0.9%	100.0%	
Middle school (1st grade)	Boys	2019	Frequency	325	1471	1770	646	65	4277	χ^2^ = 154.321 df = 4 *p* < 0.001
			Percentage	7.6%	34.4%	41.4%	15.1%	1.5%	100.0%	
		2021	Frequency	217	1289	2076	1067	127	4776	
			Percentage	4.5%	27.0%	43.5%	22.3%	2.7%	100.0%	
	Girls	2019	Frequency	389	2096	1485	223	15	4208	χ^2^ = 178.181 df = 4 *p* < 0.001
			Percentage	9.2%	49.8%	35.3%	5.3%	0.4%	100.0%	
		2021	Frequency	286	1900	1901	507	40	4634	
			Percentage	6.2%	41.0%	41.0%	10.9%	0.9%	100.0%	
Middle school (2nd grade)	Boys	2019	Frequency	379	1571	1556	628	68	4202	χ^2^ = 344.394 df = 4 *p* < 0.001
			Percentage	9.0%	37.4%	37.0%	14.9%	1.6%	100.0%	
		2021	Frequency	279	1174	2330	1054	229	5066	
			Percentage	5.5%	23.2%	46.0%	20.8%	4.5%	100.0%	
	Girls	2019	Frequency	338	1948	1475	301	30	4092	χ^2^ = 226.981 df = 4 *p* < 0.001
			Percentage	8.3%	47.6%	36.0%	7.4%	0.7%	100.0%	
		2021	Frequency	195	1770	2180	581	55	4781	
			Percentage	4.1%	37.0%	45.6%	12.2%	1.2%	100.0%	
Middle school (3rd grade)	Boys	2019	Frequency	480	1837	1743	553	72	4685	χ^2^ = 251.844 df = 4 *p* < 0.001
			Percentage	10.2%	39.2%	37.2%	11.8%	1.5%	100.0%	
		2021	Frequency	249	1345	1919	906	33	4452	
			Percentage	5.6%	30.2%	43.1%	20.4%	0.7%	100.0%	
	Girls	2019	Frequency	314	1967	1827	386	29	4523	χ^2^ = 144.489 df = 4 *p* < 0.001
			Percentage	6.9%	43.5%	40.4%	8.5%	0.6%	100.0%	
		2021	Frequency	130	1693	2013	570	46	4452	
			Percentage	2.9%	38.0%	45.2%	12.8%	1.0%	100.0%	

## Data Availability

The data presented in this study are available upon request from the authors. The data are not publicly available owing to privacy and ethical restrictions.
